# A novel augmented reality-based surgical guidance system for total knee arthroplasty

**DOI:** 10.1007/s00402-021-04204-4

**Published:** 2021-10-26

**Authors:** Sandro F. Fucentese, Peter P. Koch

**Affiliations:** 1 University Hospital Balgrist, University of Zurich, Forchstrasse 340, 8008 Zurich, Switzerland; 2 Department of Orthopaedic Surgery and Traumatology, Kantonsspital Winterthur, Brauerstrasse 15, 8401 Winterthur, Switzerland

**Keywords:** Augmented reality, Accuracy, Balance, Ligaments, Computer-assisted surgery, TKA

## Abstract

**Introduction:**

Many of the functional complications that arise after total knee arthroplasty (TKA) are caused by a non-optimal balance of the knee after surgery. Over the past 20 years, technology has been used in the Operating Room (OR) to help improve precision and balance. The results of Computer-Assisted Surgery (CAS) and robotic systems show improved accuracy regarding implant positioning but a relatively small improvement in patient-reported outcomes and implant survival compared to conventional TKA. Recently, Augmented Reality (AR) has been proposed as a technology that could improve accuracy in orthopaedic surgery, providing a more efficient and cost-effective solution.

**Materials and methods:**

This article describes a novel AR-based surgical guidance system that measures intra-operatively the effect of prosthesis alignment and positioning on soft tissue balance. The system is integrated in a pair of smart glasses and two small sensors and displays surgical targets directly in the field of view of the surgeon.

**Results:**

The system has been used in a limited number of cases. While the preliminary experience has been positive, clinical research is ongoing to confirm to confirm the performance of the system and the impact on clinical outcomes.

**Conclusion:**

Augmented Reality can be a valuable tool to improve accuracy in TKA. The use of smart glasses and integrated sensors improves the efficiency of the procedure, particularly when coupled with single-use instrumentation. A novel protocol for soft tissue assessment allows for a 3-dimensional evaluation of the ligaments and a better measurement of the effect of tibial rotation.

## Introduction

There is a widespread interest in improving total knee arthroplasty (TKA) outcomes in the orthopaedics community. While studies have demonstrated that TKA is a procedure that can be performed with increasing safety [1–3] and with excellent longevity [4], the literature shows that approximately 20% of TKA patients express some dissatisfaction about the results of their surgery [5, 6].

Some studies have even reported that over 40% of patients are unsatisfied with their lifestyle after the operation [7]. This percentage is likely to be higher in the subgroup of younger, more active people that already accounts for 45% of TKA patients [8].

Patient satisfaction is certainly multifactorial: several aspects contribute to the outcomes of a TKA procedure, including the implant design, proper individual target definition and patient expectations, but precision and accuracy in implant alignment and soft tissue balance are inherently desirable. Over the past 20 years, technology has been used in the Operating Room (O.R.) to help improve precision and balance. However, the results of Computer-Assisted Surgery (CAS) in Knee Replacement have not invariably proven to be better than those obtained using conventional technique in terms of survivorship, function or satisfaction [9–12].

In recent years, robotic-assisted solutions have been introduced into the orthopaedic market in a further attempt to improve outcomes with the aid of technology. However, robots are largely based on the same underlying technology that has been in use in Computer-Assisted Surgery since the early 2000s, and still require external cameras as well as additional hardware. This raises the question of what impact such technology has on the efficiency in the O.R., both in terms of logistics and cost [13].

To date robotic solutions in joint replacement have not been proven to significantly improve outcomes [14]. While rigorous, independent studies may still reveal improved outcomes with electronic technologies, any claimed benefits would have to be assessed in relation to the costs associated with their achievement.

The cost of robotic systems can be over $1 million. In particular, a previous study estimated a total capital investment for one of the most common robotic system of $1.362 million, considering a cost of $934,728 over 5 years with an additional 10% per year for 2–5 years for the associated service contract and a 3% discount rate. Considering 100 cases/year over a 5-year period, they estimated an additional cost of around $2700 per procedure [15]. This amount did not include the cost of the consumables such as proprietary drapes, pins and reflective markers which can further increase the cost per procedure. Maintenance costs must also be included for contracts that provide for the loan of the robot and, in this case, hospitals might incur higher costs for the disposables. The cost per procedure increases as the number of cases per year decreases. As most robotic systems are limited to using proprietary implants, hospitals that have made large capital outlays may be significantly tied to the companies offering them, and therefore, much less able to negotiate favourable implant pricing in the future.

The evidence to date does not seem to clearly justify the considerable increases in costs associated with the use of robotic systems in joint replacement. In fact, early results of modern robotic systems show similar trends to those of CAS TKA, with improved accuracy and consistency regarding implant positioning but a relatively small improvement in Patient-Reported Outcome Measure (PROM) and implant survival compared to conventional TKA [16].

This may partly be due to the fact that precise and accurate alignment was aimed at targets that were not ideal for the specific patients in which they were used. The focus is, therefore, more and more on techniques that deliver patient-specific alignment and balance.

Patient-specific surgical instruments have also been proposed as an alternative technology to improve accuracy while also improving the O.R. efficiency and limiting the associated cost of the procedure [17]. Despite having proved to achieve results comparable to navigation in terms of accuracy according to several studies [18, 19], this technology presents the inherent limitation of relying on a preoperative plan and allowing only limited intraoperative adjustment based on the assessment of the soft tissues, which is not available at the planning stage. In addition, results are not consistent across different systems and studies reported in the literature [20, 21].

More recently, Augmented Reality (AR) has generated an increasing interest as a technology that could improve accuracy in orthopaedic surgery and specifically in knee replacement, providing a more efficient and cost-effective solution compared to robotic-assisted surgery. AR is an interactive experience of a real-world environment, where the objects that reside in the real world are enhanced by computer-generated information, as opposed to Virtual Reality, which completely replaces the real-world environment with a simulated one.

Although it has not yet been widely adopted in orthopaedics, AR has shown the potential to be a time-saving, risk-reducing, and accuracy-enhancing technology in orthopaedic surgery [22].

In particular, this article focuses on a novel augmented reality-based surgical guidance system which intra-operatively measures the effect of prosthesis alignment and positioning on soft tissue balance (NextAR TKA, Medacta International SA, Castel San Pietro, Switzerland).

## System overview

NextAR TKA was cleared by the US FDA and by the Australian TGA in 2020 and to the authors’ knowledge, it is the first AR-based guidance system to be officially cleared for use in TKA in these markets. The system was also CE marked in the first half of 2021. Applications of the same AR platform for shoulder and spine surgery are also available in Europe and USA.

**Fig. 1:**
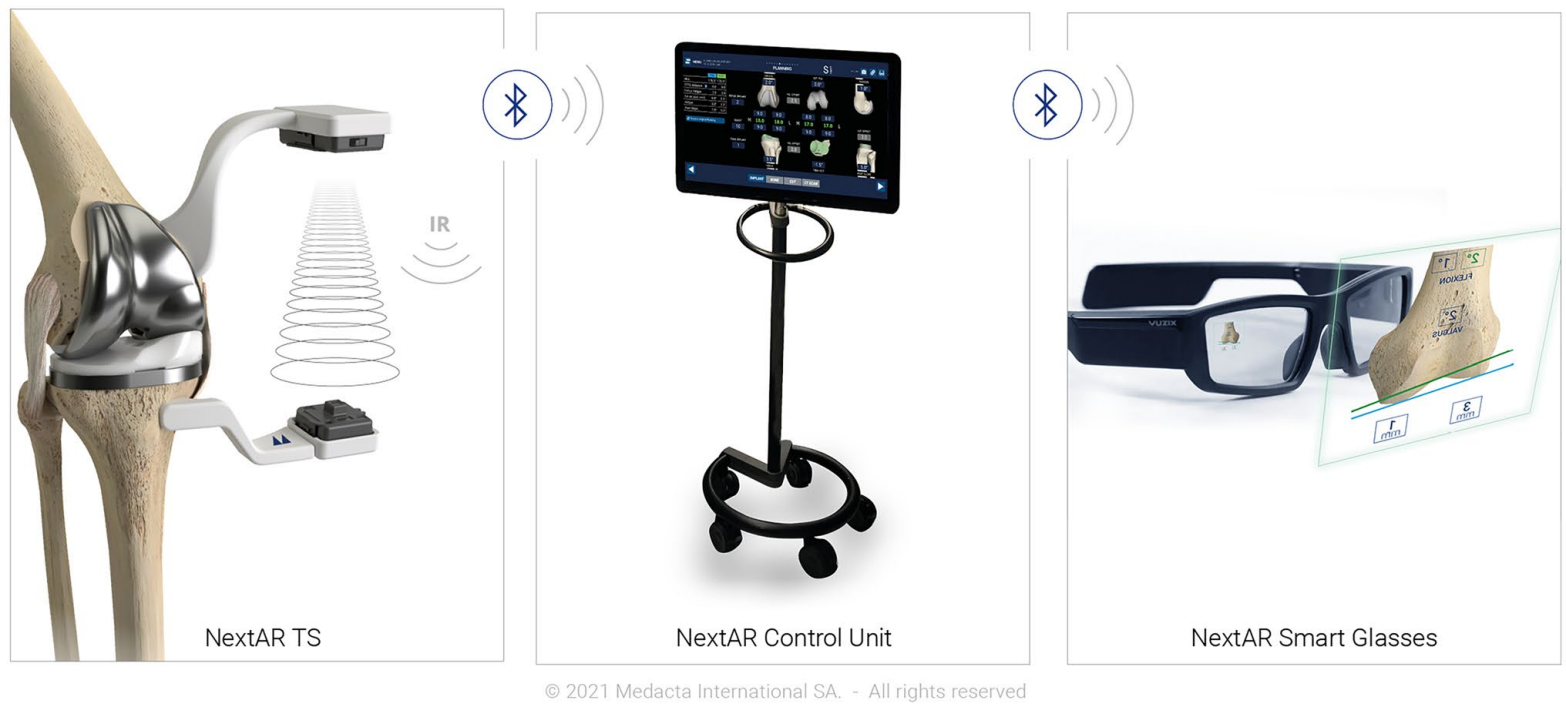
Components of the NextAR platform: single-use IR sensors (left), control unit (middle), and smart glasses (right)

The footprint of the system is minimal, since it comprises only a pair of smart glasses, two small single-use sensors and a control unit (Fig. 1). The same hardware is also used for the other applications of the platform for shoulder and spine surgery.

In the AR systems, the information flow is displayed to the user via either a standard screen (typically a hand-held device with an integrated camera) or a so-called “head-mounted display”. The latter is used in the system described in this review in the form of a pair of smart glasses. This device offers additional benefits to the user, as it allows for real-time visualisation of the relevant information directly in the field of view, while performing surgical actions. This improves the user experience, as well as hand–eye coordination [23]. The smart glasses are equipped with an integrated battery and are fully wireless, for an improved usability in the O.R. The glasses are light-weight, and the information is displayed in a simple interface, which in the authors’ experience did not cause any fatigue and headache even after prolonged use.

The information displayed in the smart glasses is collected by a pocket-sized wireless optical tracking system. The system is integrated in an active infrared camera and an active tracker and does not require the use of external cameras, thus eliminating any line-of-sight issues. The two sensors are provided terminally sterile and single use, and they are certified for over 4 h of battery life. They can also be replaced at any point during the procedure (e.g. if they are inadvertently dropped) without the need to recalibrate the system. When the tracker is placed in the correct measurement zone, it transmits its spatial position in six degrees of freedom (3 translations, 3 rotations) with an error ≤ 0.5°/0.5 mm, as indicated in the instruction for use of the device. The system warns the user whenever the tracker approaches the boundaries of the recommended transmission zone to avoid loss of accuracy. The camera and the tracker can be fixed to the femur and tibia within the surgical incision, which eliminates the need to use percutaneous bone pins, and can be clipped to the instruments used throughout the procedure to provide guidance to the surgeon through the smart glasses.

The hardware is completed by a control unit which is connected via Bluetooth to the tracking systems and the smart glasses. The control unit receives the information from the sensors and runs the guidance software. After the initial setup, the control unit is only needed if the surgeon decides to modify the surgery plan, as every other step can be controlled and visualised through the sensors and smart glasses.

The NextAR TKA system is cleared for use with GMK Sphere medially stabilised knee (Medacta International SA, Castel San Pietro, Switzerland).

## Ligament assessment

Many of the functional complications that arise after TKA are caused by a non-optimal balancing of the knee after surgery [24, 25]. The system described in this review uses a novel approach to intra-operatively measure the effects of prosthesis alignment and positioning on soft tissue balance. Specifically, it provides a visual representation of the strain of the collateral ligaments throughout the flexion range of the knee. Unlike other systems available on the market, the strain of the collateral ligaments is measured by tracking their origins and insertions in 3D, and not as an indirect measurement of the medial and lateral gaps.

Preoperatively, the patient-specific origins and insertions of the medial collateral ligament (MCL) and lateral collateral ligament (LCL) are identified on a CT scan, using a proprietary semi-automated algorithm based on a validated protocol described in the literature, which relies on the virtual palpation of a standard set of points on the bone [26]. This information is used to track the ligaments in real time in the O.R.

Before any bone resection, the resting length of the collateral ligaments can be acquired with the knee in full extension, to be used as a reference for the rest of the procedure. Compensating for cartilage wear while acquiring the resting length can provide an indication of the native length of the collateral ligaments, assuming that no permanent, disease-related change has happened over time [27]. This base pattern is then used as reference target during the whole surgery and can be checked as much as needed, and can be used to modify the planning even before any bone resection.

This novel approach allows for a 3-dimensional assessment of the effect of each planning parameter on ligament contracture and elongation. In particular, the effect of tibia rotation can be properly evaluated, which is not possible through an indirect measurement of the medial and lateral gaps. Indeed, if the gaps remain stable but the tibia rotates, the length of the collateral ligaments changes. However, this element is often overlooked since guidance systems that rely exclusively on bone tracking cannot capture this effect.

**Fig. 2:**
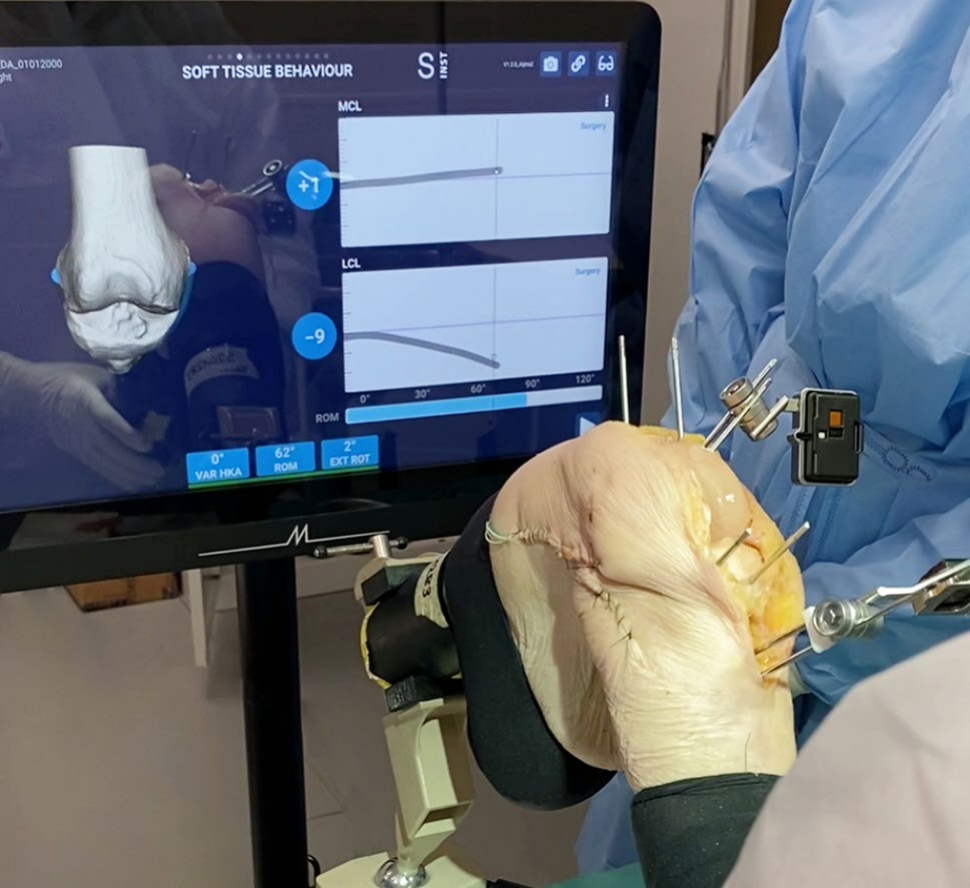
The length of the MCL (top chart) and the LCL (bottom chart) throughout the range of motion can be measured and displayed by the system at any point during the surgery

A qualitative assessment of the measurements obtained with the system on cadaveric specimens, exemplified in Fig. 2, has confirmed that the results are in line with the pattern of collateral ligament elongation measured in vivo for the same implant in a published study, with a shortening of the LCL with increasing knee flexion and a more isometric behaviour of the MCL [28].

## Case workflow

The system uses computer tomography (CT) scans of the hip, knee and ankle for preoperative planning and intraoperative bone registration. CT scans should be performed according to the protocol validated by the manufacturer to guarantee the necessary accuracy in the 3D reconstruction and proper evaluation of the alignment.

### Preoperative planning

Cases are managed through a secure cloud-based web-portal. Before each surgery, the surgeon creates a new case. The CT scans are then uploaded to the same portal directly by the radiology team. An engineer from the company reconstructs a 3D model of the patient’s bones and creates a preliminary plan according to the surgical preferences indicated by the surgeon on his account. Mechanical Alignment and Kinematic Alignment are the default planning protocols on the platform, but the surgeon has the option to adjust his preference for each parameter. During the planning phase, the company engineer identifies the origins and insertions of the collateral ligaments to be used intra-operatively to assess the soft tissues, as described in Sect. 3. The plan is submitted to the surgeon, who can review it in a 3D web-based planning tool and may change any parameters, evaluating the effects in real time. Once validated, the plan can be downloaded for the surgery directly from the case page on the web-portal and loaded onto the system.

### Surgical procedure

First, the holders for camera and tracker are pinned to the bone within the surgical incision. Femur and tibia are then registered acquiring 30 points on each bone. The surgeon is guided through the process as the points to be registered are progressively displayed in the smart glasses. In the author’s experience, the registration process takes approximately 3–4 min. An automatic perturbation algorithm runs in the background to stress the model and ensure the robustness of the registration.

**Fig. 3:**
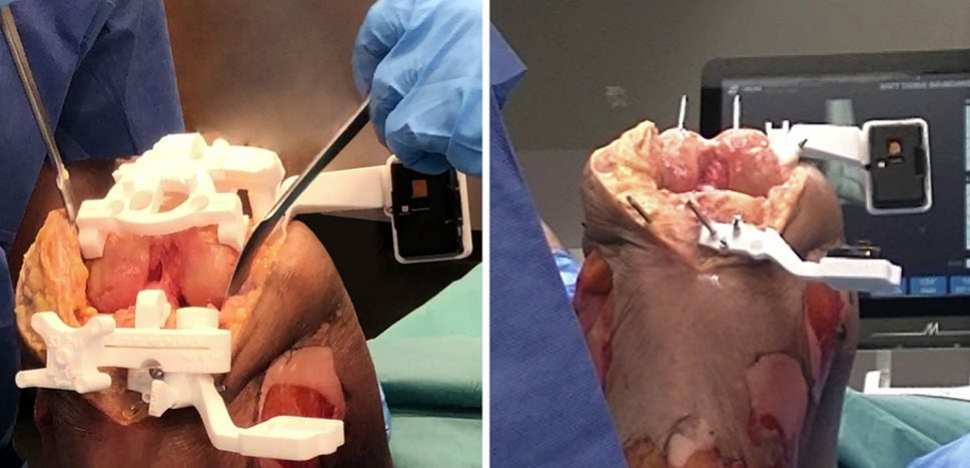
Patient-specific holders for the camera and the tracker are positioned on the bone through dedicated guides (left) and are left in place once the guides have been removed (right)

Alternatively, patient-specific surgical guides (MyKnee, Medacta International SA, Castel San Pietro, Switzerland) can be used to position the holders for the camera and tracker and register the bone in a single step. Once the guide is positioned on the bone, a fast four-point algorithm ensures the correct positioning of the guide and completes the registration of the bone. The holders for the camera and tracker are connected to the patient-specific guide and can be left in place once the guide is removed (Fig. 3).

The surgeon can then proceed with the ligament assessment as described in Sect. 3. The additional information on the soft tissues can be used to modify the preoperative plan before proceeding with the bony resections. The complete preoperative plan can be reviewed intra-operatively, including the possibility to navigate the CT scans with the implant superimposed onto the bone.

**Fig. 4:**
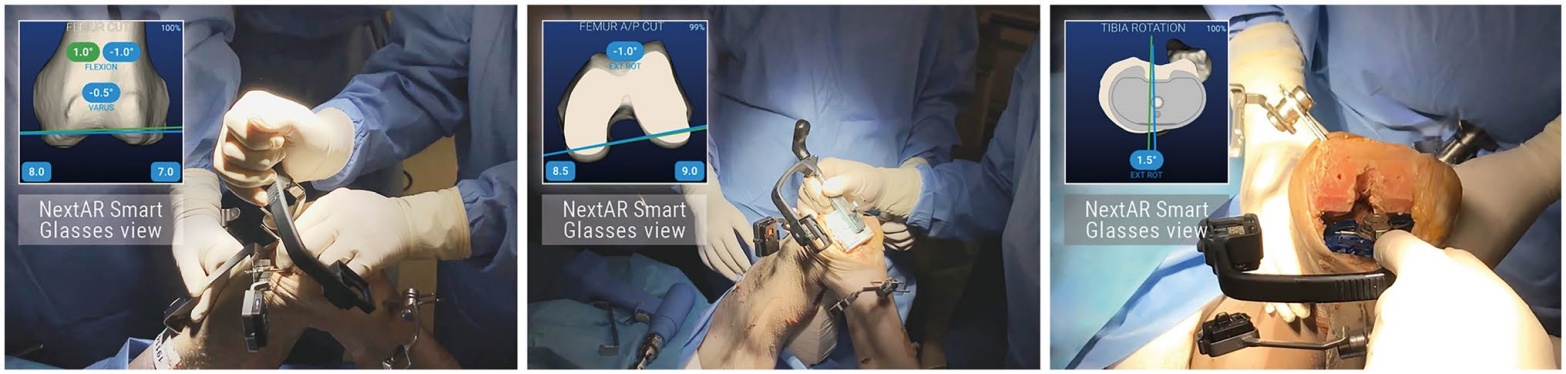
Examples of how the AR system guides the surgeon in the positioning of the distal femoral cut block (left), 4 in 1 block (middle) and tibial component (right)

The surgeon can set the order of the resections according to his standard practice. As shown in Fig. 4, the AR system guides the surgeon through the positioning of the cutting guides, as the position of the instruments with respect to the surgery plan is displayed in real time in the smart glasses. The system also helps to achieve the target rotation of the tibial component, which is crucial for proper balance since it influences the elongation of the collateral ligaments, as described in Sect. 3.

After the trial implant have been positioned on the bones, the elongation pattern of the ligaments can be acquired to test the stability of the joint and compared this with the initial assessment to confirm that the desired target has been achieved.

## OR efficiency

The system described in this review has a very compact footprint and does not require external cameras and dedicated operators.

**Fig. 5:**
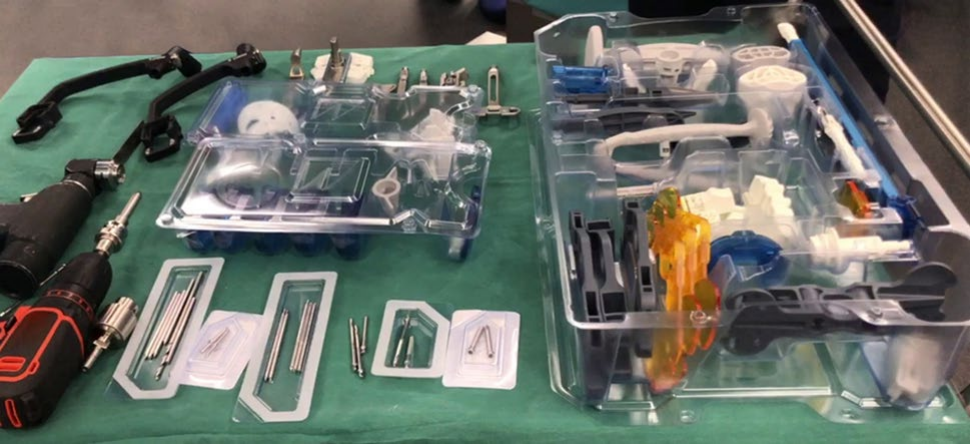
Single-use and reusable instruments required for a total knee arthroplasty with NextAR

Moreover, it is compatible with a complete single-use instrument set (GMK Efficiency, Medacta International SA, Castel San Pietro, Switzerland), which is delivered terminally sterile and has been found to be as accurate as metal instruments according to previous studies [29, 30]. In addition to the AR-specific hardware, only one generic tray, one size-specific femur tray and one size-specific tibia tray are needed, since the size of the implant components are known from the preoperative plan (Fig. 5). A previous study analysed intraoperative size changed with the MyKnee system (which follows the same preoperative steps as the system described in this review) on 602 components and reported only a 2% size change on the femur and 8.8% on the tibia [18]. A backup is always available in case of size changes.

A study that modelled OR turnover time, tray sterilisation, tray management time, and 90-day infection rates for 200 sites and 500 cases per site found that the median total cost savings with single-use instruments was $994 per case. The largest driver for cost savings was tray sterilisation. In cases where single-use instruments were used, up to 51% of operating days could have accommodated an additional procedure due to the time savings in OR turnover [31]. Similar benefits can be expected when the single-use instruments are used in combination with the AR system, which adds only a small tray of dedicated reusable instruments and does not require significant setup time.

## Future steps

At the time of writing, the system has been used in a limited number of cases in Europe, Australia and the United State. While the preliminary experience has been positive, further clinical experience must be collected to confirm the full potential of the system. In particular, the direct assessment of ligament elongation might represent a step forward in terms of soft tissue balancing, but more data must be collected to create a reliable baseline, to be used to better interpret the values recorded intra-operatively and adjust the surgery plan accordingly.

Several cadaver tests performed by the authors and other surgeons involved in the development have confirmed the accuracy of the system. However, clinical research data must be collected to confirm the performance of the system and the impact on clinical outcomes. The system is currently being investigated within a clinical study which was granted approval by the Western Australia South Metropolitan Health Service Human Research Ethics Committee. In addition, the authors are involved in a premarket clinical study in Switzerland.

As part of this clinical investigation, the authors will also evaluate the feasibility of using a validated biomechanical model of the knee presented in a previous study [32], combined with a kinematic model of the specific implant used with the system, for the purpose of predicting the effect of each surgical parameter on ligament strain. The initial ligament assessment performed intra-operatively will be used to feed patient-specific information to the model and generate a patient-specific output. This innovative assessment would provide further guidance to the surgeon in defining the best target to achieve proper balance with the potential of a significant impact on clinical outcomes.

Applications of the same technology platform for shoulder and spine surgery have also been introduced on the market recently, while applications for partial knee replacement and hip replacement have already been announced by the manufacturing company as part of the development pipeline. In principle, this would allow hospitals and clinics to manage most cases with one single system, thus reducing the capital investment and the logistics burden to provide technology solutions for different specialties.

## Conclusions

This review has described a novel AR-based surgical guidance system for TKA with the potential to improve surgical accuracy and soft tissue balance by feeding real-time information to the surgeon directly in his or her field of view, which helps to define and achieve the best implant position and limb axis.

A novel protocol for soft tissue assessment allows direct real-time measurement of the strain of the collateral ligaments throughout the range of motion by tracking the movement of their origins and insertions. This allows for a 3-dimensional evaluation of the ligaments and a better measurement of the effect of tibial rotation.

The system is integrated in a pair of smart glasses and two small sensors, which eliminate the need for an external camera and improve the efficiency of the procedure, particularly when coupled with single-use instrumentation.

Clinical research is ongoing to confirm the performance of the system and the impact on clinical outcomes.
